# Biological and clinical significance of radiomics features obtained from magnetic resonance imaging preceding pre-carbon ion radiotherapy in prostate cancer based on radiometabolomics

**DOI:** 10.3389/fendo.2023.1272806

**Published:** 2023-10-20

**Authors:** Guangyuan Zhang, Zhenshan Zhang, Yulei Pei, Wei Hu, Yushan Xue, Renli Ning, Xiaomao Guo, Yun Sun, Qing Zhang

**Affiliations:** ^1^ Department of Radiation Oncology, Shanghai Proton and Heavy Ion Center, Fudan University Cancer Hospital, Shanghai, China; ^2^ Shanghai Key Laboratory of Radiation Oncology, Shanghai, China; ^3^ Shanghai Engineering Research Center of Proton and Heavy Ion Radiation Therapy, Shanghai, China; ^4^ Department of Research and Development, Shanghai Proton and Heavy Ion Center, Fudan University Cancer Hospital, Shanghai, China

**Keywords:** metabolomics, radiomics, pre-carbon ion radiotherapy, prostate cancer, biochemical recurrence (BCR)

## Abstract

**Introduction:**

We aimed to investigate the feasibility of metabolomics to explain the underlying biological implications of radiomics features obtained from magnetic resonance imaging (MRI) preceding carbon ion radiotherapy (CIRT) in patients with prostate cancer and to further explore the clinical significance of radiomics features on the prognosis of patients, based on their biochemical recurrence (BCR) status.

**Methods:**

Metabolomic results obtained using high-performance liquid chromatography coupled with tandem mass spectrometry of urine samples, combined with pre-RT radiomic features extracted from MRI images, were evaluated to investigate their biological significance. Receiver operating characteristic (ROC) curve analysis was subsequently conducted to examine the correlation between these biological implications and clinical BCR status. Statistical and metabolic pathway analyses were performed using MetaboAnalyst and R software.

**Results:**

Correlation analysis revealed that methionine alteration extent was significantly related to four radiomic features (Contrast, Difference Variance, Small Dependence High Gray Level Emphasis, and Mean Absolute Deviation), which were significantly correlated with BCR status. The area under the curve (AUC) for BCR prediction of these four radiomic features ranged from 0.704 to 0.769, suggesting that the higher the value of these four radiomic features, the greater the decrease in methionine levels after CIRT and the lower the probability of BCR. Pre-CIRT MRI radiomic features were associated with CIRT-suppressed metabolites.

**Discussion:**

These radiomic features can be used to predict the alteration in the amplitude of methionine after CIRT and the BCR status, which may contribute to the optimization of the CIRT strategy and deepen the understanding of PCa.

## Introduction

Prostate cancer (PCa) is the second most prevalent form of cancer among males and accounts for approximately 7% of newly diagnosed cancer cases worldwide (1, 2). PCa is a significant contributor to cancer-related mortality. Globally, annual deaths surpass 350,000 with over 1.2 million new cases diagnosed each year (2–4).

Radiation therapy (RT) is a curative treatment option for localized PCa, with outcomes comparable to surgery (5). Local tumor control is positively associated with the RT dose (6). A particle beam is considered an ideal modality for RT because of its ability to facilitate better dose distribution through the Bragg peak and its relatively high biological efficacy resulting from high linear energy transfer (7). However, the high radiation dose delivered to prostate tumors is limited by the radiation tolerance of the bladder and rectum.

A retrospective study conducted across multiple institutions included 2157 patients who underwent carbon ion radiotherapy (CIRT). The findings demonstrated remarkable tumor control with minimal adverse effects. The five-year survival rate without biochemical recurrence (BCR) was 92%, 89%, and 92% for patients categorized as low-, intermediate-, and high-risk, respectively. Notably, no Grade 3 toxicities were observed in the study population (8). However, several patients experience local recurrence or distant metastasis following CIRT. Furthermore, prognostic prediction methods after CIRT, particularly BCR prediction, have seldom been investigated. Posttreatment pathological changes are useful for response evaluation and treatment plan optimization. However, unlike during surgery, it is difficult to collect pathological information after CIRT. Therefore, imaging is an important approach to evaluate the irradiation response.

Magnetic resonance imaging (MRI) and prostate-specific membrane antigen positron emission tomography (PSMA-PET) play pivotal roles in detecting PCa. Radiomics is a high-throughput qualitative analysis method of these imaging modalities that can provide traditional morphological information, and also a pack of image signatures related to the pathological profile of the lesion. Radiomics has been applied for differential diagnosis, risk classification, radiation planning, and prognostic prediction in PCa studies (9). Recent studies have shown that models based on pre-treatment MRI radiomics features have good predictive performance for BCR (10–13). These results indicate that radiomics is a potential tool for predicting the prognosis of PCa treatment.

Although radiomics features contain prognostic information, their underlying biological significance is not well known. Multiomics, similar to radiogenomics, is a bridge connecting radiomics and molecular pathology. Several studies have highlighted the relationship between imaging features and genetic profiles (14) and have provided multidimensional insights into tumor aggressiveness and progression (15). Metabolomics is another powerful tool to demonstrate the biological status of cancer, as the metabolic reprogramming phenomenon of tumors plays an essential role in tumor initiation, development, and recurrence (16, 17). PCa is a typical metabolism-related disease, and the androgen receptor drives metabolic reprogramming that promotes prostate cancer growth (18). In particular, the analysis of metabolite alterations in urine samples reflects biological characteristics and predicts the prognosis of PCa, thereby avoiding additional injury caused by biopsy (19–21). Similarly, our previous metabolomic study reported that metabolites in urine, such as arginine, glutamine, and histidine, were significantly downregulated after CIRT (22), suggesting that CIRT could repress tumor metabolism and consequently contribute to the effective control of PCa. However, its efficacy in optimizing prognosis requires further investigation.

Therefore, the potential that radiomic features contain with respect to prognosis, such as BCR, which could be related to metabolite alterations post-CIRT, evoked our interest. A combination of metabolomics and radiomics could be a feasible, non-invasive, and informative method that could functionally and morphologically explain the underlying biological significance of radiomics.

In this study, we aimed to explore the biological significance of radiomic features using correlation analysis between metabolites significantly downregulated by CIRT and MRI radiomic features pre-CIRT. Thus, we explored and verified the clinical and biological significance of these radiomic features in a cohort, along with BCR outcomes.

## Materials and methods

### Patients and CIRT

Between July 2014 and December 2022, 450 patients with pathologically confirmed prostate adenocarcinoma were treated with CIRT at our center. 63 consecutive patients staged as T2-3N0M0 according to the American Joint Committee on Cancer TNM Staging System and Prognostic Groups for Prostate Cancer (AJCC 8th edition) were enrolled in this analysis. 37 patients were selected based on the availability of pre-CIRT MRI images and pre- and post-CIRT urine samples to conduct a correlation analysis between radiomics and metabolomics. Another 26 patients were enrolled to verify the clinical significance; 13 patients experienced BCR, whereas the other 13 patients with similar clinical characteristics did not experience BCR. All 26 patients had pre-CIRT MRI images, which enabled us to conduct a correlation analysis between radiomics and BCR status. Patient characteristics are presented in **Table 1**.

**Table 1:** Clinical characteristics of the participants.

Characteristics	overall
T stage, n (%)	
T2	50 (79.4%)
T3	12 (19%)
T4	1 (1.6%)
Prognosis group, n (%)	
I	1 (1.6%)
II	43 (68.3%)
III	19 (30.2%)
Gleason score, n (%)	
≤7	39 (61.9%)
>7	24 (38.1%)

### CIRT

All 63 patients underwent CIRT according to the institutional research protocol. CIRT was delivered using the Siemens IONTRIS particle therapy device, and delineation of the clinical target volume (CTV) was based on the ESTRO ACROP consensus (23). The CTV for CIRT included the entire prostate and partial seminal vesicles based on different risk groups. For intermediate-risk patients, the CTV encompassed the entire prostate gland and the bottom 1–1.5 cm of the seminal vesicles. For high- and very high-risk patients, the CTV included the entire prostate gland and the bottom 2–2.5 cm of the seminal vesicles.

CIRT was administered using the Siemens IONTRIS particle therapy system. The prescription dose was determined using the Syngo planning system (V13B, Siemens, Germany), considering the local effect model 1 and accounting for the relative biological effectiveness (RBE)-weighted dose. To ensure accurate treatment delivery, bladder and rectal preparations were performed before each treatment.

The irradiation doses were 59.2 Gy (RBE) to 66 Gy (RBE) delivered in 16–24 fractions. 13 patients received a simultaneous integrated boost for the visible lesion identified on both PSMA PET/computed tomography (CT) and MRI with a prescription dose of 72 Gy (RBE) delivered in 16 fractions to the gross tumor volume (GTV). At least two associate professors identified and contoured the GTV based on both PSMA PET/CT and MR images. For the treatment planning, the prescribed dose should cover 100% of the CTV at a minimum of 95%, while 100% of the planning target volume should have at least 90% coverage by the isodose line.

### Metabolomics of interest identification

#### Reagents

Ultrapure water was obtained from Millipore Milli-Q Integral 5 Ultrapure Water System (Billerica, MA, USA). LC-MS-grade acetonitrile and methanol were purchased from Shanghai Cancer Rehabilitation Club (SCRC, Shanghai, China). Ammonium hydroxide and formate was purchased from Merck (Darmstadt, Germany). All standard compounds were obtained from Tansoole (Shanghai, China), Thermo Fisher Scientific (Waltham, MA, USA), Merck Aladdin (Shanghai, China), and Macklin (Shanghai, China).

#### Urine sample collection and preparation

Urine samples were collected from patients within 4 h preceding administration of the initial fraction and within 4 h after completion of the final fraction. After collection, the samples were immediately stored at 4°C. To eliminate bacterial contamination, a 0.22 µm membrane filter was used. For each thawed sample, 80 μL of urine was mixed with 160 μL of acetonitrile/methanol (1:1, v/v) in a 1.5 mL tube under an ice bath. After vortexing for 30 s, the mixtures were ultrasonicated in an ice bath for 10 min. The tube was then stored at −20°C for at least 1 h. After centrifugation (12000 rpm, 15 min) at 4°C, 200 μL of the supernatant was transferred to a sample vial insert (250 μL) at 4 °C, awaiting injection of LC-MS. Pooled quality control (QC) samples were prepared by combining equal volumes (5 µL) of the supernatant from each sample.

#### High-throughput UPLC-MS/MS analysis

The metabolomic data of the PBMC samples were collected using a UPLC system (ExionLC™ 2.0, AB SCIEX, USA) coupled to a quadrupole time-of-flight mass spectrometer (X500B, AB SCIEX, USA). An ACQUITY UPLC BEH Amide Column (130 Å, 1.7 µm, 2.1 mm × 100 mm; Waters, USA) was used for metabolite separation in both positive and negative modes. The water phase (A) was prepared by mixing 25 mmol of ammonium hydroxide and 25 mmol of ammonium formate with 1 L of water, and the organic phase (B) was acetonitrile. The total liquid flow rate was 0.3 mL/min, and the gradient was set as follows: 0–1 min: 95% B, 1–14 min: 95% B to 65% B, 14–16 min: 65% B to 40% B, 16–18 min: 40% B, 18–18.1 min: 40% B to 95% B, and 18.1–23 min: 95% B. The injection volume was 2 μL, and the oven column temperature was maintained at 40°C.

In the analysis queue, all samples were tested using a random injection sequence. Mass axis calibration, blank sample injection, pooled QC injection, and laboratory mixed standard compound solution injection were gradually performed every eight sample injections. The electrospray ionization source parameters were as follows: ion source gas 1 (55 psi), ion source gas 2 (55 psi), curtain gas (35 psi), temperature (600°C), and spray voltage (5500 V or −4500 V in positive or negative modes), respectively. For TOF MS, the scan parameters were mass scan range (50–1000 Da), accumulation time (0.2 s), declustering potential (60 V or −60 V in positive or negative modes, respectively), and collision energy (10 V or −10 V in positive or negative modes, respectively). MS/MS spectrum acquisition was triggered according to the information-dependent acquisition mode with a maximum of 10 candidate ions, and dynamic background subtraction was performed. For TOF MS/MS, the scan parameters were as follows: mass scan range (25–1000 Da), accumulation time (0.05 s), collision energy (35 or −35 V in positive or negative modes, respectively), and collision energy spread (15). For the other items, the default settings were retained.

Raw data files (.wiff2 and.wiff.scan) were converted to mzXML and mgf formats using ProteoWizard with a peak-picking filter to the centroid. The peak relative quantitative matrices of all samples were obtained using our large-scale metabolomics data processing software based on the R language (software copyright registration number:2023SR0256527). All metabolite peaks were annotated with MetDNA2 (http://metdna.zhulab.cn/), and statistically significant unknown peaks were further annotated with the aid of available reference standards in our laboratory and web-based resources, including the Human Metabolome Database (HMDB) (http://www.hmdb.ca/), Massbank (http://www.massbank.jp), and PubChem (pubchem.ncbi.nlm.nih.gov).

### Radiomics analysis

#### MR imaging

MR was performed prior to the first CIRT fraction. The MRI protocol comprised T2WI, DWI, and T1-weighted contrast-enhanced series. The detailed imaging parameters are listed in **Table 2**. All patients underwent the same imaging protocol using a 3T scanner (MAGNETOM Skyra, Siemens Healthcare, Erlangen, Germany).

**Table 2:** Magnetic resonance imaging parameters.

Sequence	TE (ms)	TR (ms)	Slice thickness/gap	Matrix	FOV (cm^2^)	b Value
Axial T2WI	89	9040	3.5/0	320*256	200	–
Axial T1 vibe	1.39	3.56	3/0.6		350	–
Axial DWI	66	6680	3/0	116*116	200	50,1000,1500

FOV, field of view; T2WI, T2 weighted imaging; DWI, diffusion weighted imaging.

#### Volume of interest segmentation and radiomics features extraction

We manually segmented the entire prostate on axial T2WI slice-by-slice. All segmentations were performed by a radiologist with 8 years of experience in uranological tumor diagnosis who was blinded to the clinical and pathologic information.

Radiomics features were extracted using the Radiomics module’s built-in 3D slicer (version 4.10.2, www.slicer.org). We extracted 14 shape, 18 first-order, and 75 texture features, which are available at https://pyradiomics.readthedocs.io/en/latest/.

#### Radiomics features screening based on reliability analysis

15 cases were randomly selected for radiomic feature reliability analysis. The voiding delineation of these cases was repeated by one radiologist at two time points with seven-day intervals. The intraclass correlation coefficient (ICC) of the radiomic features at the two time points was then calculated. Radiomics features with an ICC > 0.900 were considered reliable and included in further analysis.

#### Statistics

All statistical metabolic analyses were performed using MetaboAnalyst 5.0 (https://metaboanalyst.ca/), R (version 3.6.3), and MedCalc (version 18.2.1). A volcano plot consisting of fold change (FC) analysis and the Wilcoxon signed-rank test was used to identify significantly different metabolites (p<0.05, |FC|>2). The Pearson correlation coefficient was calculated and visualized using the complexHeatmap package in the R software (version 3.6.3). Radiomics features from different BCR statuses were compared using *t*-tests. Receiver operating curve (ROC) analysis was performed to evaluate the prognostic value of the radiomics features. The significance level was set at p<0.05.

## Results

### Correlation between metabolomics and radiomics

#### Inhibition effect of CIRT on localized PCa metabolism

**Figure 1:**
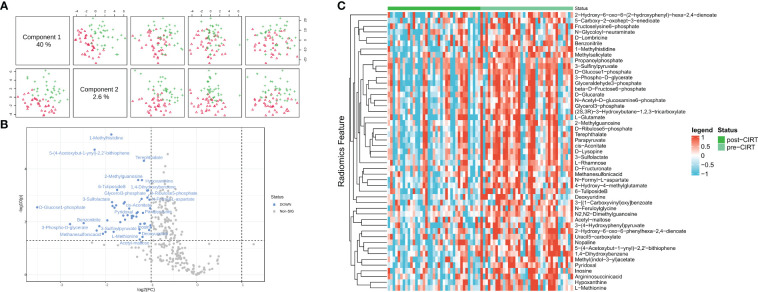
Inhibition effect of CIRT on localized PCa metabolism. The repression effect of CIRT on PCa. Samples were clearly divided into groups **(A)**. The landscape of the top 50 altered metabolites; red shows the group post-CIRT and green shows the group pre-CIRT **(B)**. A volcano plot shows the significantly downregulated 39 metabolites after CIRT **(C)**. CIRT, carbon ion radiotherapy.

A total of 284 peaks were identified in the patients’ urine samples by comparing the metabolic changes before and after CIRT. PCa exhibited a distinct difference between pre- and post-CIRT samples after normalization and paired analysis, indicating that the metabolite profiles were considerably altered after CIRT (**Figure 1A**). 49 metabolites showing significant changes were observed in the volcano plot (P<0.05, |FC|>2). These metabolites were all downregulated, demonstrating that CIRT effectively inhibited or delayed the progression of PCa metabolism (**Figure 1B**). Additionally, the heat map displays the landscape of the 49 metabolites that displayed considerable alterations in the two groups (**Figure 1C**). the significantly altered metabolites were list in **Supplementary Table 1**.

### Metabolism enrichment analysis

**Figure 2:**
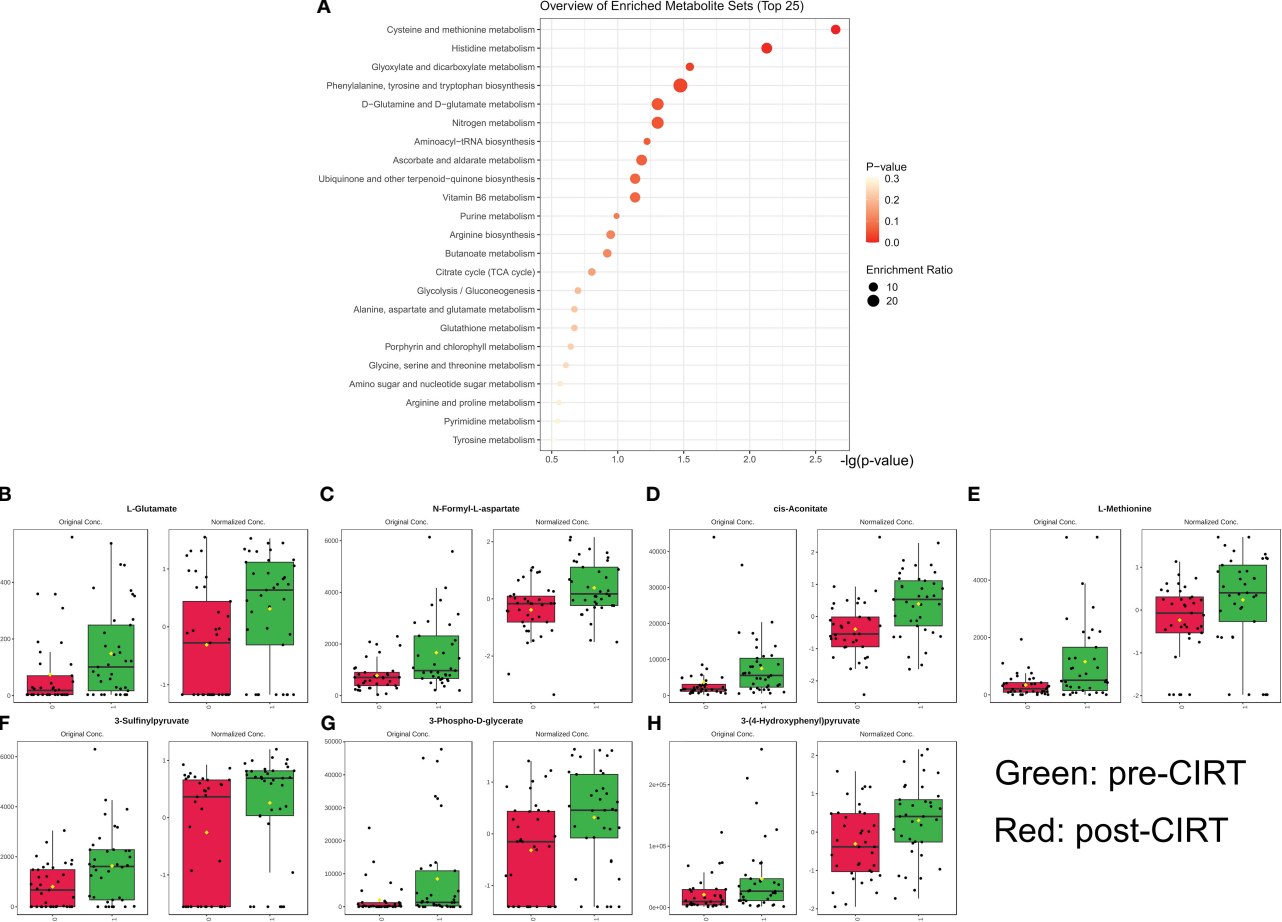
Metabolism pathway enrichment analysis. Pathway enrichment analysis and core metabolites. Based on 38 significantly downregulated metabolites, 6 pathways were significantly enriched (p<0.05) **(A)**. The alteration landscape of overlapped 7 genes in 6 pathways hits; green indicates pre-CIRT and red indicates post-CIRT **(B–H)**. CIRT, carbon ion radiotherapy.

Significantly altered metabolites were predominantly enriched in pathways involving cysteine, methionine, histidine, glyoxylate, dicarboxylate, phenylalanine, tyrosine, tryptophan, glutamine, glutamate, and nitrogen (**Figure 2A**) (P<0.05). Seven of the 49 altered metabolites were associated with these pathways, including methionine, 3-sulfinylpyruvate, 3-phospho-D-glycerate, glutamate, N-formyl-L-aspartate, cis-aconitate, 3-4-hydroxyphenyl, and pyruvate. These seven metabolites were regarded as the key metabolites representing typical CIRT effects (**Figures 2B–H**) (y-axis labels represent peak intensity). the enrichment analysis results were list in **Supplementary Table 1**.

### Reliability of radiomics features

The mean ICC of 107 radiomics features was 0.987 with a 95% confidence interval of 0.981–0.994. Three radiomic features—sphericity with a mean ICC of 0.855, SizeZoneNonUniformityNormalized with a mean ICC of 0.799, and Small Area Emphasis with a mean ICC of 0.784—were excluded from further analysis because they did not achieve an ICC value of 0.900.

### Correlation between core metabolites and radiomics features

**Figure 3:**
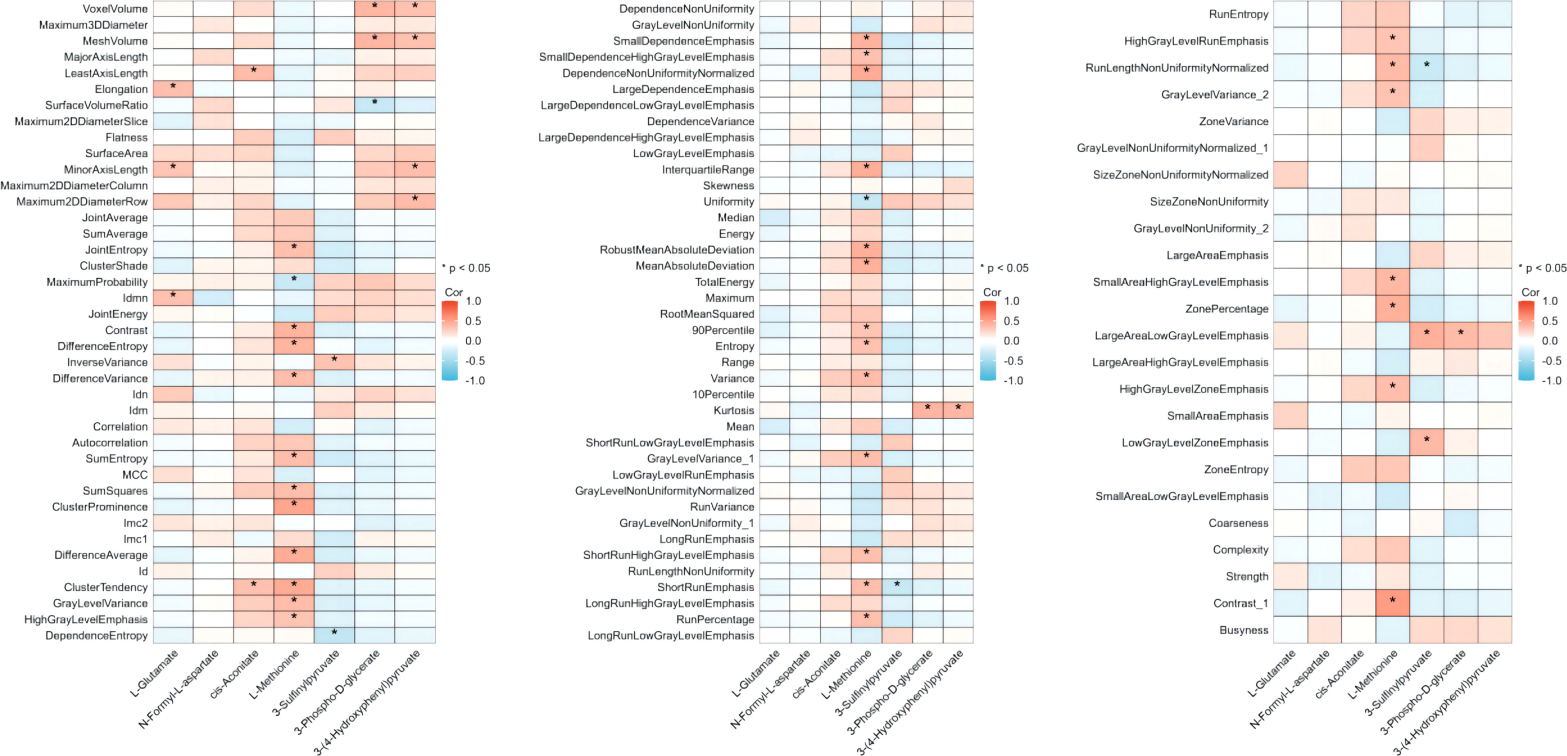
Correlation between core metabolites suppressed by CIRT and pre-CIRT T2WI radiomics features. Correlation between the value of 104 radiomics pre-CIRT and the AEs of 7 metabolites. Red indicates positive correlation and blue indicates negative correlation. CIRT, carbon ion radiotherapy; T2WI, T2-weighted imaging.

The alteration extent (AE) of the metabolites was calculated as post-value+0.001/pre-value+0.001. The correlation coefficient and p-value between the radiomic features and the AE of each core metabolite were calculated and are shown in **Figure 3**.

**Figure 4:**
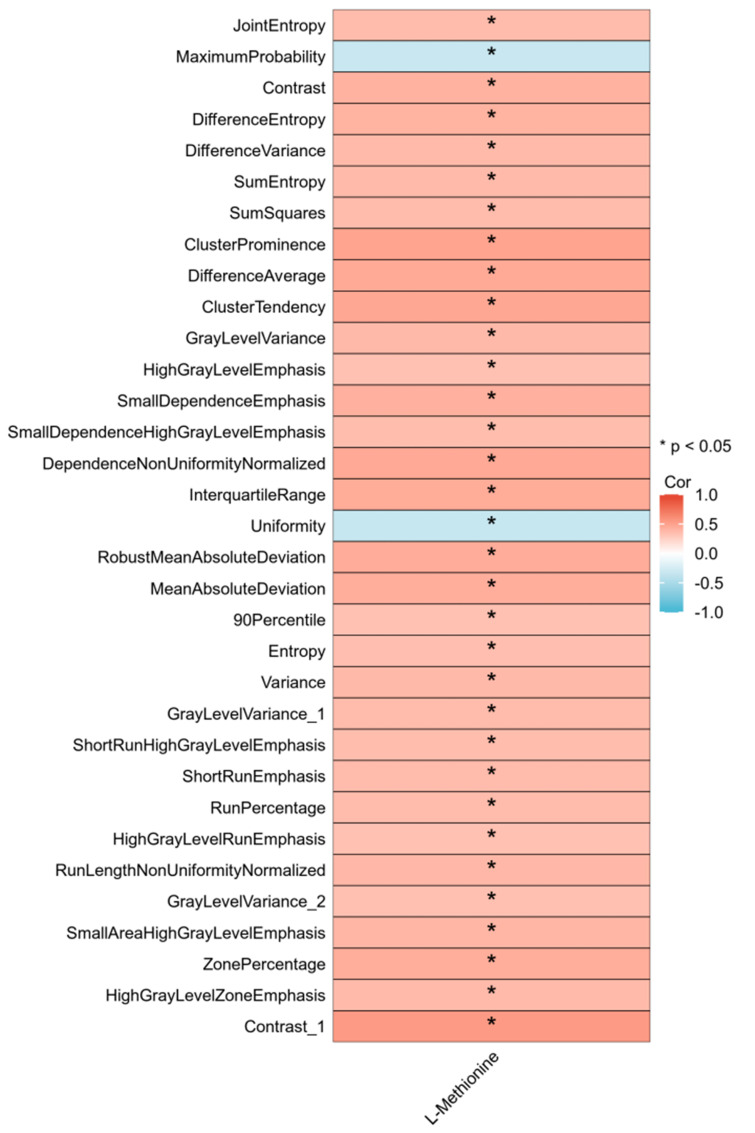
Thirty-three radiomics features related to alteration extent (AE) of L-Methionine. Correlation between the value of 33 radiomics pre-CIRT and the AEs of 7 metabolites. Red indicates positive correlation and blue indicates negative correlation. CIRT, carbon ion radiotherapy.

In total, 33 of 104 radiomic features were significantly correlated with the extent of methionine alteration. The median correlation coefficient was 0.376 (95% confidence interval [CI], 0.366–0.389). The correlation between the 33 radiomic features and the extent of methionine alteration is illustrated in **Figure 4**.

### Prognostic value of CIRT-suppressed metabolites related to radiomics features

**Figure 5:**
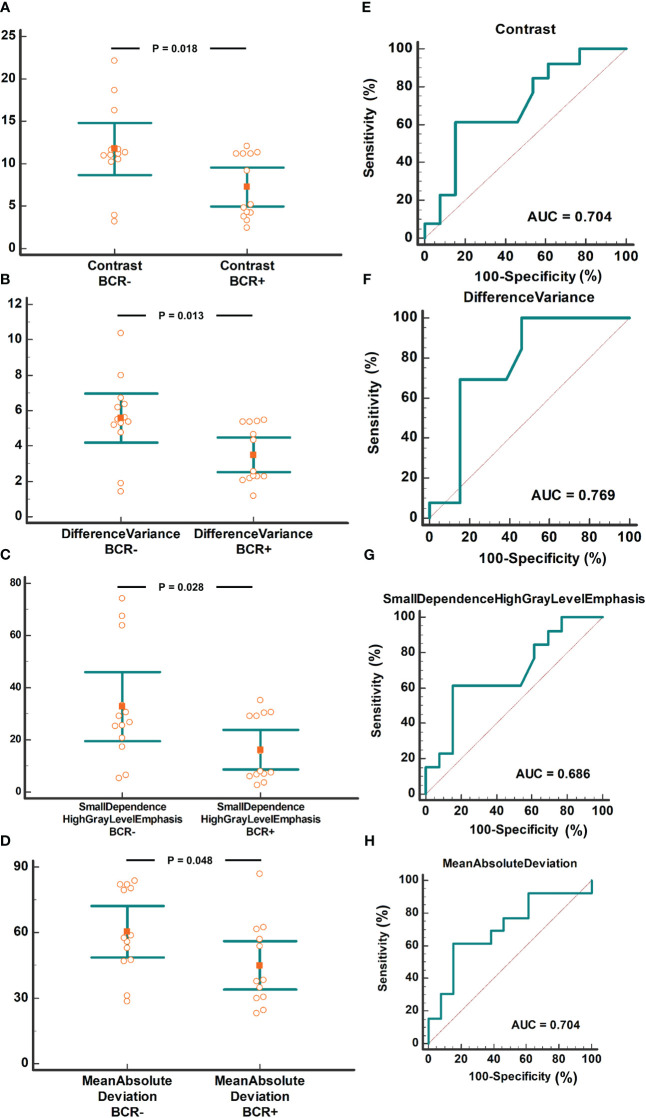
Radiomics features of the four CIRT-suppressed metabolites compared based on BCR status. The comparison of contrast between the BCR+ and BCR- groups with AUC analysis **(A/E)**. The comparison of Difference Variance between the BCR+ and BCR- groups with AUC analysis **(B/F)**. The comparison of Small Dependence High Gray Level Emphasis between BCR+ and BCR- groups with AUC analysis **(C/G)**. The comparison of Mean Absolute Deviation between BCR+ and BCR- group with AUC analysis **(D/H)**. CIRT, carbon ion radiotherapy; BCR, biochemical recurrence; AUC, area under the curve.

14 of the 33 radiomic features based on the metabolomic cohort were not identified in the radiomics cohort. Therefore, 19 radiomic features were analyzed to determine their relationship with BCR. The values of the four radiomics features, Contrast, Difference Variance, Small Dependence High Gray Level Emphasis, and Mean Absolute Deviation, were significantly lower in cases of biochemical recurrence. A detailed comparison of these radiomics features is presented in **Table 3**. The area under the curve (AUC) of these four radiomics features ranged from 0.704 to 0.769. A value comparison based on the BCR status and ROC curves is shown in **Figure 5**.

**Table 3:** Four CIRT-suppressed metabolites related to radiomics features and their comparison.

Radiomics features	BCR-		BCR+		
	Mean	SD	Mean	SD	P value
Contrast	11.729	5.125	7.243	3.757	0.018
DifferenceVariance	5.585	2.283	3.494	1.616	0.013
SmallDependenceHighGrayLevelEmphasis	32.773	22.097	16.263	12.438	0.028
MeanAbsoluteDeviation	60.478	19.513	44.935	18.442	0.048

## Discussion

CIRT, as an effective modality of radiotherapy, has played a crucial role in PCa treatment; however, the optimal selection of the population and an effective marker for prognosis prediction remain elusive and warrant investigation. Given that images of MRI and urine samples from PCa are convenient and non-invasive and could effectively reflect the biological characteristics of PCa, we performed the present study to comprehensively analyze these two methods.

Radiomic features from MRI are considered powerful imaging biomarkers for BCR prediction. Based on the radiomics features from biparametric MRI before radical prostatectomy or radiation, the predictive model for BCR showed an AUC of 0.84 and 0.73 in the training and validation cohorts, respectively (12). In another study, an AUC of 0.63 was obtained in the prediction of BCR using pre-radiation radiomics features from T2WI (10). These values were comparable to our results (AUC of 0.704 to 0.769).

Metabolic dysregulation is an essential component of PCa initiation, development, and progression (24, 25). Therefore, for subsequent analysis, we first analyzed the metabolic alterations caused by CIRT; 49 of 124 metabolites were significantly downregulated post-CIRT. In the analysis of pathway enrichment, cysteine and methionine metabolism, histidine metabolism, glyoxylate and dicarboxylate metabolism, phenylalanine, tyrosine, and tryptophan biosynthesis, glutamine and glutamate metabolism, and nitrogen metabolism pathways were significantly enriched, which have been reported to be involved in cancer development (26–31), including PCa (32–35). After extraction of the same metabolites from the hits of the pathway enrichment analysis, seven metabolites were identified as core metabolites: methionine, 3-sulfinylpyruvate, 3-phospho-D-glycerate, glutamine, N-formyl-L-aspartate, cis-aconitate, and 3-4-hydroxyphenyl pyruvate. According to recent investigations, methionine and glutamine contribute to prostate progression (36–38) and photon RT resistance (39).

In the correlation analysis of 104 radiomic features extracted from the MRI images and seven core metabolites, a positive connection between 33 features and methionine was observed, suggesting that decreased levels of methionine post-CIRT were significantly related to the radiomic features of pre-CIRT. Moreover, investigations have revealed that a common characteristic of cancer cells is high methionine dependence (37, 40, 41) and methionine restriction has been proposed as an avenue for cancer therapy (42). For instance, in animal models of rhabdomyosarcoma (43), Yoshida sarcoma (44), hepatoma (45), and colorectal carcinoma (46), methionine restriction represses tumor growth, prevents cell migration and invasion, and prolongs patient survival. Furthermore, preclinical and clinical studies have demonstrated that the combination of methionine restriction and chemotherapeutics is effective in tumor therapy (47–49). Moreover, study also indicated the impacts of methionine on anti-tumor immune function, its downstream mechanisms on specific immune activity under radiotherapy are also worthy exploring. As for PCa, methionine related biological activity has been reported to facilitate tumor development by participating in methylation (50) and could be influenced by SNHG3 (51). Hence, the 33 pre-CIRT radiomic features reflecting the decline in methionine levels have the potential to predict the prognosis of patients with PCa treated with CIRT.

To further confirm their clinical value, these features were used to determine prognosis analysis, and we found that four pre-CIRT T2WI radiomics features were related to BCR, with AUCs varying from 0.704 to 0.769. Combined with the above results, higher values of the four features resulted in a greater amplitude of methionine decline. In addition, two out of four radiomics features identified in our study were reported previously in other prognosis research. Shiradkar et al. (12) found out that differential variance was related to BCR after either surgery or radiation. In another study, contrast extracted from pre radiation CT images was found to be with prognostic value of method free survival (11). However, the other two radiomics features were not found in previous studies. This discrepancy may arise from different biological mechanism of CRIT.

In contrast to previous studies, the segmentation reliability in the present study was much higher. The mean ICC of the 107 radiomics features was 0.987. There were 104 (97.20%) radiomic features with an ICC > 0.90. Previous studies have reported that the percentage of ICC > 0.90 varied from 9.6% to 14% (52, 53). The higher reliability in the current study may have arisen from the use of the same MR scanner, imaging protocol, and one radiologist performing the segmentation. Furthermore, although diffusion weighted imaging was another significant sequence in PCa diagnosis and evaluation, this functional imaging modality was usually with blur margin of prostate, which might arise unreliable segmentation. Therefore, we analyzed T2WI images alone in current study.

The potential biological significance of radiomics features has been previously reported. Gugliandolo et al. (54) investigated the association of MRI radiomic features with cancer aggressiveness using a correlation analysis of radiomic features with risk class, T-stage, Gleason score, extracapsular extension score, and Prostate Imaging Reporting and Data System (PI-RADS v2) score. The AUC of the selected radiomic features for predicting the aforementioned clinical aggressiveness scores ranged from 0.74 to 0.94. In addition, the radiomics features associated with the Gleason score, T-stage, and PI-RADS score showed a protective effect; namely, the higher the value of radiomics features, the lower the aggressiveness of the tumor. These results explain the findings of this study. The features related to methionine alteration could predict the prognosis of PCa treated with CIRT, indicating that patients with a higher value of the four features on MRI pre-CIRT were more sensitive to CIRT and may achieve a better local response.

The present study has some limitations. First, the small sample size may have reduced the statistical power. Second, the BCR- and metabolite-related radiomic features were not from the same cohort. Although different radiomic features were excluded, other underlying biases between the two cohorts may have interfered with the results. Finally, the radiomics features of intraprostatic lesions were not considered. Since most patients in the current cohort received ADT before MRI, tumor visibility was very low. Only 15 of the 37 patients had visible lesions.

## Conclusion

In the current study, we identified BCR-related radiomics features as well as significantly altered metabolite levels, especially methionine, in patients with PCa receiving CIRT, and revealed the potential molecular significance of these radiomics features in a radiometabolomics manner, using a combination of radiomics and metabolomics. This radiometabolomics approach may act as a potential tool for investigating the underlying biological and clinical significance of radiomics features obtained from MRI preceding CIRT in patients with PCa.

## Data availability statement

The original contributions presented in the study are included in the article/**Supplementary Material**, further inquiries can be directed to the corresponding author/s.

## Ethics statement

The studies involving humans were approved by Ethics Committee of Shanghai Proton and Heavy Ion Center, Fudan University Cancer Hospital. The studies were conducted in accordance with the local legislation and institutional requirements. Written informed consent for participation in this study was provided by the participants’ legal guardians/next of kin.

## Author contributions

GZ: Writing – original draft, Writing – review & editing. ZZ: Writing – original draft, Writing – review & editing. YP: Conceptualization, Methodology, Writing – review & editing. WH: Data curation, Validation, Writing – review & editing. YX: Data curation, Formal Analysis, Resources, Writing – review & editing. RN: Project administration, Resources, Validation, Writing – review & editing. XG: Investigation, Project administration, Writing – review & editing. YS: Conceptualization, Formal Analysis, Methodology, Project administration, Software, Supervision, Writing – review & editing. QZ: Conceptualization, Funding acquisition, Investigation, Methodology, Project administration, Software, Validation, Writing – review & editing.
