# Supplementary material for: Biological and clinical significance of radiomics features obtained from magnetic resonance imaging preceding pre-carbon ion radiotherapy in prostate cancer based on radiometabolomics
Source: Front Endocrinol (Lausanne). 2023 Oct 20;14:1272806. doi: 10.3389/fendo.2023.1272806 (PMC10644841; doi:10.3389/fendo.2023.1272806)
Supplement: Supplementary file 2 [file Table_2.docx]

| Characteristics | overall |
| --- | --- |
| T stage, n (%) |  |
| T2 | 26 (70.3%) |
| T3 | 10 (27%) |
| T4 | 1 (2.7%) |
| Prognosis group, n (%) |  |
| I | 1 (2.7%) |
| II | 19 (51.4%) |
| III | 17 (45.9%) |
| Gleason score, n (%) |  |
| ≤7 | 20 (54.1%) |
| >7 | 17 (45.9%) |
