# Supplementary material for: Biological and clinical significance of radiomics features obtained from magnetic resonance imaging preceding pre-carbon ion radiotherapy in prostate cancer based on radiometabolomics
Source: Front Endocrinol (Lausanne). 2023 Oct 20;14:1272806. doi: 10.3389/fendo.2023.1272806 (PMC10644841; doi:10.3389/fendo.2023.1272806)
Supplement: Supplementary file 3 [file Table_3.docx]

| Characteristics | overall |
| --- | --- |
| T stage, n (%) |  |
| T2 | 24 (92.3%) |
| T3 | 2 (7.7%) |
| Prognosis group, n (%) |  |
| II | 24 (92.3%) |
| III | 2 (7.7%) |
| Gleason score, n (%) |  |
| ≤7 | 20 (76.9%) |
| >7 | 6 (23.1%) |
